# The Relation of Veterinary Medicine to the Public Health1Read before the Thirty-seventh Annual Meeting of the American Veterinary Medical Association at Detroit, Mich., September 5, 1900.

**Published:** 1900-09

**Authors:** William Herbert Lowe

**Affiliations:** Paterson, N. J.


### THE RELATION OF VETERINARY MEDICINE TO THE PUBLIC HEALTH.[1]

By Dr. William Herbert Lowe,
PATERSON, N. J.

I think it is a safe statement to make that people recognize and appreciate much quicker and in a fuller sense what veterinary medicine does for their pet animals and for themselves in a commercial sense in protecting the animal wealth than they do for what the science does for the health and lives of the people themselves.

[1] Read before the Thirty-seventh Annual Meeting of the American Veterinary Medical Association at Detroit, Mich., September 5, 1900.

When the veterinary practitioner is successful in restoring to health his patron's pet dog, or in saving the life of his valuable horse, his services are in the great majority of instances appreciated; but what he does to protect the health and lives of the people is seldom understood or recognized.

A great work is being done by qualified veterinary practitioners all over this broad land in sanitation and preventive medicine, and it is a deplorable fact that the nature and scope of this work is not more fully understood by otherwise intelligent people. Preventive medicine will advance as its value is recognized and demanded by the public generally.

Not many years since, when contagious pleuro-pneumonia broke out among cattle, and the animal industry of the country suffered heavy financial losses, it did not take the public long to demand State and national legislation to exterminate the disease. This disease struck the most sensitive thing in creation—the bank account—and although there was absolutely no danger of transmission of the disease to mankind, yet measures were taken at once, which, under able guidance at Washington, resulted in the extermination of the disease. While this was as it should have been, yet it seems strange from the stand-point of the veterinary profession that the public is so slow in supporting and encouraging what veterinary science is capable of doing for the health and lives of the people themselves. The conscientious veterinarian is constantly looking out to improve sanitary conditions, have animals kept under hygienic conditions, as well as to treat diseased animals, and last, but not least, to prevent the transmission of disease to human beings. It should be remembered that the veterinarian is truly the guardian of the public health, as in many instances diseases transmissible to man as well as bad sanitary conditions first come under his observation. If he deals with them as they should be dealt with he protects human life and health. Is it not better to prevent infection than to allow it to occur and then try to relieve or cure the patient? Of course, it is not intended or contemplated to put the human practitioner out of business, but simply to relieve the busy practitioner as much as possible.

Speaking seriously, permit me to say that a large integral part of preventive medicine belongs essentially to comparative medicine, and nobody recognizes and appreciates this fact more than far-seeing members of that time-honored profession.

The truth is that the two branches of medicine, human and veterinary, are inseparable. No advance can be made in one without benefiting the other. The contemporary advances in medicine have necessitated an extensive remodelling of the medical and veterinary curricula and methods, and we see as a result the incorporation of the principal veterinary colleges in our great universities.

No fact has drawn a closer connection between animal and human diseases, or the protection of the public health through domestic animals, than the introduction of the antitoxin principles of serum therapy. The immunizing of animals against fatal contagious diseases; the rapid and certain diagnosis of latent diseases which cannot be known by physical symptoms, and their early isolation before they have spread their disease germs among their kind, or even to the human family; the cure of some diseases by the administration of repeated doses of attenuated virus, and the prevention of the development of disease by their early administration, are phases of the rôle of veterinary science which make it a great preserver of the public health. Contemplate for a moment the importance of these serums: Tuberculin, used for the diagnosis of tuberculosis in dairy cattle; mallein, used for the diagnosis of glanders in the horse; vaccine, employed to protect mankind from smallpox; tetanus antitoxin, employed in the treatment of lockjaw; diphtheria antitoxin, employed in the treatment of diphtheria; as well as the antitoxins employed in anthrax, rabies and other diseases. The value of animal serums employed for diagnostic, prophylactic and therapeutic purposes to mankind cannot be estimated.

We hear a good deal in these latter days about "the passing of the horse" which is absurd; but even if such were the case there would still remain a vast field open in this country for qualified veterinarians in connection with the work of State and local boards of health, throughout the length and breadth of this broad land, to say nothing of laboratory and experimental work which veterinarians must participate in, if true science is to advance as it should. Another great field for the veterinarian is in aiding the development and maintenance of the animal industry and wealth of this country; not only in presenting and actually treating and restoring sick animals to health, but primarily in the science of breeding, and in the development of animal life for special purposes useful to man as well as in securing a sound, wholesome meat and milk supply. Then, again, people will always have dogs and pet animals that they want cared for when ill, so it must be evident to every thoughtful person that the field of veterinary research and practice is almost illimitable to the scientific veterinarian.

The United States Bureau of Animal Industry is doing a great work in the matter of the inspection of meat for interstate and foreign trade, but this matter of meat-inspection should not stop here. We as a people are a business people, and here again the financial considerations seem to have more weight with us than that of our health. We are more particular, yes, far more particular, about the quality and healthfulness of the meat we export to England, France and Germany than we are of the meat produced for home consumption.

This is no criticism of the work of the Bureau of Animal Industry, for this branch of the Federal service is carried out as far as the law contemplates and in a very effectual and far-reaching manner. It is the function of local boards of health to take care of this matter. Competent veterinarians must be selected to do this work if it is to be of any real value to the public.

The scandals and sensational reports of bad meat supplied to our soldiers in the Spanish-American war must have suggested the importance of an adequate veterinary inspection of animal food and what it would have meant to the health and lives of the men serving in our army.

A great deal has been said recently about milk-inspection, but it seems to me that the authorities in a great many instances begin at the wrong end of this whole business. Instead of beginning by inspecting the milk, as is often done, they should begin by inspecting the animals, their stables, and the dairy generally. Every dairy should be under veterinary supervision. A good cow, healthy and sound, properly stabled and properly fed is going to give good, wholesome milk; and a poor cow, unthrifty or unhealthy, improperly stabled and improperly fed is going to give poor, unwholesome milk.

The veterinary inspector should require good hygienic and sanitary conditions. The cows as well as the stables should be kept clean, the drainage and ventilation must be looked after, and the cows must have ample air, space and light. Proper and good food and pure drinking water are essential.

The milk as soon as drawn from the cows should be removed to another building, to be taken care of and prepared for market, away from objectionable odors and injurious bacteria and germs. Not infrequently healthy milk absorbs poisonous or disease germs between the time it is drawn from the cow and the time it is delivered to the consumer. A few enterprising firms have done much to provide a wholesome milk supply, such as the Fairfield Dairy Company in my own State; but the general way cows are cared for and fed, and the way that milk is usually handled, is far from what it should be. The public need educating in this matter, and local boards of health should require proper veterinary supervision over dairies and the milk supply in every locality.

I do not wish to tire you with a long paper, so will conclude with the request that the relation of veterinary medicine to public health shall be discussed as its importance demands.

